# The Effect of Different Suture Removal Time Intervals on Surgical Wound Healing

**Published:** 2006-10-01

**Authors:** Masoud Parirokh, Saeed Asgary, Mohammad Jafar Eghbal

**Affiliations:** 1 *Department of Endodontics, Dental School, Kerman University of Medical Sciences, Kerman, Iran*; 2 *Department of Endodontics, Iranian Center for Endodontic Research, Dental School, Shahid Beheshti University of Medical Sciences, Tehran, Iran*; 3 *Department of Endodontics, Iran Center for Dental Research, Dental School, Shahid Beheshti University of Medical Sciences, Tehran, Iran*

**Keywords:** Periapical Surgery, Suture Removal, Wound Healing

## Abstract

**INTRODUCTION:** This study was carried out to compare the effect of different suture removal time on surgical wound healing.

**MATERIALS AND METHODS:** Twenty-one male albino rabbits were used. Under general and local anesthesia a moucoperiosteal rectangular flap was raised in each animal and then repositioned and sutured. The animals were randomly divided into three experimental groups of seven animals each. In group I and II the sutures were removed after 3 and 5 days respectively and were followed up for 7 and 14 days after surgery. In group III the sutures were removed after 7 days and were followed up for 14 days after surgery. Tissue reactions were observed and recorded using inflammation and gingival indexes at 7 and 14 days after surgery in all three groups. Inflammation and gingival indexes were analyzed by Kurskal Wallis, Firedman and Wilcoxone tests.

**RESULTS:** Results showed that inflammation index was significantly different with two other groups at the day 7 after surgery (*P*<0.008). Gingival index in group II was significantly different from two other groups at the day 14 (*P*<0.028); however, there was no significant difference between group II and III at the same interval.

**CONCLUSION:** Based on result of this study, 5 days was recognized to the best time interval for suture removal in comparison with two other time intervals.

## INTRODUCTION

Periapical surgery is a current treatment in endodontics which has many indications such as failed nonsurgical endodontic treatment that has irretrievable root filling material or intraradicular post, canal calcification, exploratory surgery, and procedural errors such as instrument fragmentation, root perforation, and symptomatic overfilling (1).

Mucoperiosteal flap is usually employed for periapical surgery and suturing will be used for closure of surgical wound (2).

The objective of suturing is to place various layers of tissues in close contact, so that a minimal quantity of new connective tissue will be required to restore the structural integrity of the tissue during the minimum possible time. A variety of suture materials are currently used in surgery within the mouth, including organic and synthetic non-resorbable and resorbable materials, amongst them monofilament sutures are the most acceptable ones (1,3,4).

Sutures placed after surgery are partly embedded in tissue and partly bathed in saliva with a mean concentration of approximately 750 million bacteria per milliliter. The inflammation caused by these bacteria produces erythema surrounding the puncture wounds and leads clinicians to suspect that the suture could wick the bacteria into the surgical site itself (5).

It has been shown that intraoral sutures placed produce a tissue response that is distinctly different from the response observed at other experimental sites; this response is a result of the presence of moisture and infectious potential with a consequent tendency towards rapid epithelial invagination (3).

It has been shown that a perisutural epithelial sleeve develops after 3 days and can enrobe the entire suture track after 7 days. An intense inflammatory response to suture materials and the trauma of suture placement is visible after 3 days (3).

The healing capacity of oral tissues is excellent. It has been shown that epithelial streaming as a sheet or as fingers is observed after 2 days, eventually resulting in a multilayered seal (6,7). After 4 days an epithelial barrier has been formed (8).

Many researchers believed that suture removal after surgery should be performed as soon as possible because of their concern about unreasonable effect on wound healing by plaque accumulation on suture materials (4,8,9); however, others believe that suture removal should be delayed until healing has been established in surgical site (10-13). The difference between researchers, ideas regarding suture removal time has caused a controversy among them. It has been advised to remove suture material at 2 days to 7 days after surgery by different authors.

Therefore, the purpose of this study was to compare the effect of different suture removal time intervals on surgical wound healing.

## MATERIALS AND METHODS

The research protocol was approved by the Research Ethics Committee of Kerman University of Medical Sciences. Twenty-one adult male albino rabbits weighting 2.5–3 kg were used. All experimental procedures were completed after intra-peritoneal injection of 7.5 mg/ kg ketamine HCl (Alfasan, Woerden, the Netherlands) and 0.1 mg /kg xylazine (Alfasan). After anesthesia, the head and neck area of the animals were scrubbed with betadine iodine (Daropakhsh, Tehran, Iran) and their mouths rinsed with 0.12% chlorhexidine gluconate solution (Sharedaru, Tehran, Iran) mouthwash. An infiltration injection of 2% lidocaine (Daropakhsh, Tehran, Iran) with 1:80000 epinephrine was then made in the anterior areas of each rabbit mandible where operation was performed. Then a full mucoperiosteal rectangular flap was made and reflected as bone was exposed. The flap was reflected for 5 minutes and then was sutured by 4/0 PVDF (CG, Tehran, Iran). The animals were placed on soft diet until the end of the experiment.

The animals were randomly divided into three experimental groups of seven animals each. In group I and II the sutures were removed after 3 and 5 days respectively and were followed up for 7 and 14 days after surgery. In group III the sutures were removed after 7 days and were followed up for 14 days after surgery. Each time interval for all animals the surgical area was observed and a check list for gingival and inflammation indexes modified from Masse et al (1993) study (14) with following criteria was filled.

***A) Inflammation Index:***

 0 = without redness and having normal appearances

+1 = mild inflammation: limited redness

+2 = moderate inflammation: redness and edema with hypertrophy of gingival tissues

+3 = sever inflammation: redness, edema with hypertrophy of gingival tissues, and spontaneous bleeding

***B) Gingival Index:***

+1 = very poor: without epithelium at the incisional edges, pus and infection, bleeding in palpation, more than 50% of gingival tissue is red

+2 = poor: granulation tissue formation at incisional edge, bleeding in palpation, connective tissue exposure without covering epithelium, more than 50% of gingival tissue is red

+3 = good: without bleeding in palpation, granulation tissue, and connective tissue exposure, 50% of gingival tissue is red

+4 = very good: without bleeding in palpation, granulation tissue, and connective tissue exposure, 25% of gingival tissue is red

+5 = excellent; pinkish gingiva, without bleeding in palpation, granulation tissue, and connective tissue exposure

All check lists were filled by an endodontist who was blind to suture removal time intervals.

Inflammation and gingival indexes were analyzed by Kruskal Wallis, Wilcoxone and Friedman tests.

**Figure 1:**
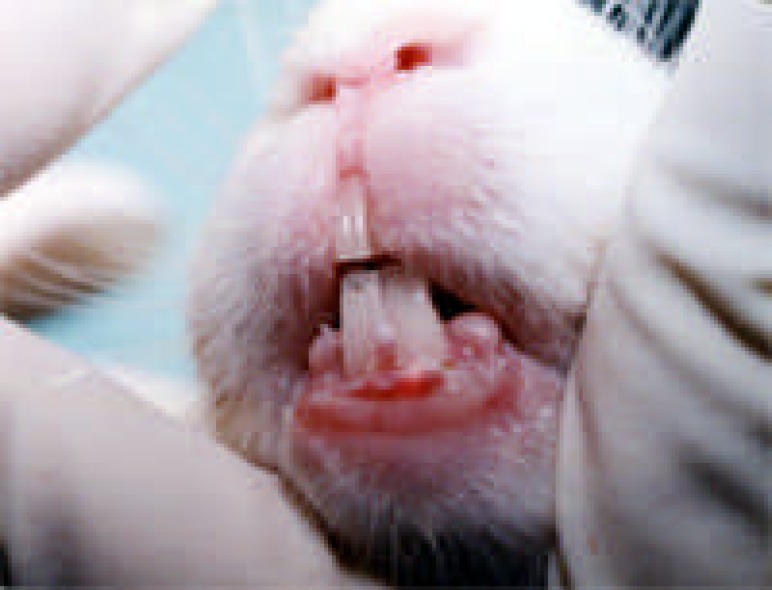
Clinical view of flap at time of suture removal (3 days group).

**Figure 2:**
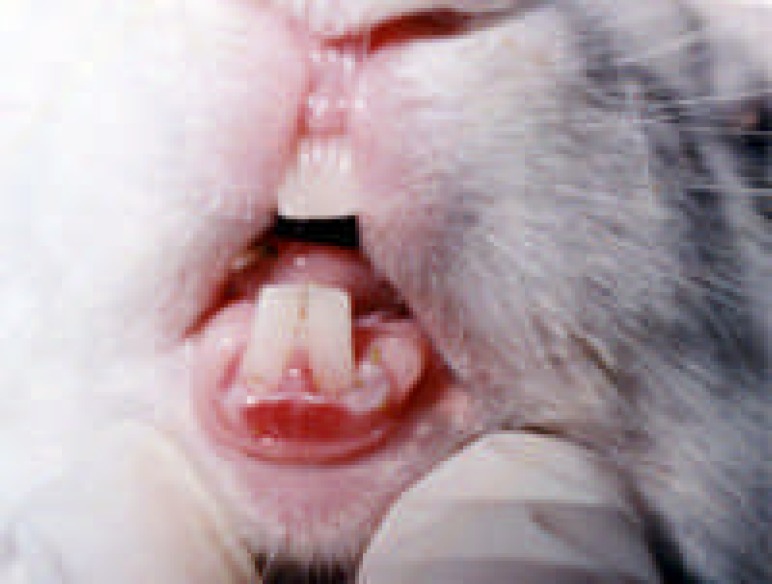
Seven days after surgery (3 days group).

## RESULTS

**Figure 3:**
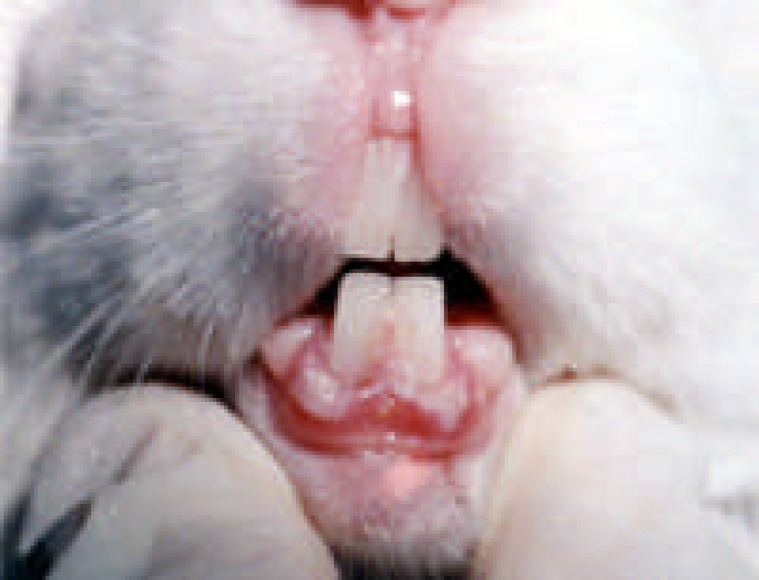
Fourteen days after surgery (3 days group).

**Figure 4:**
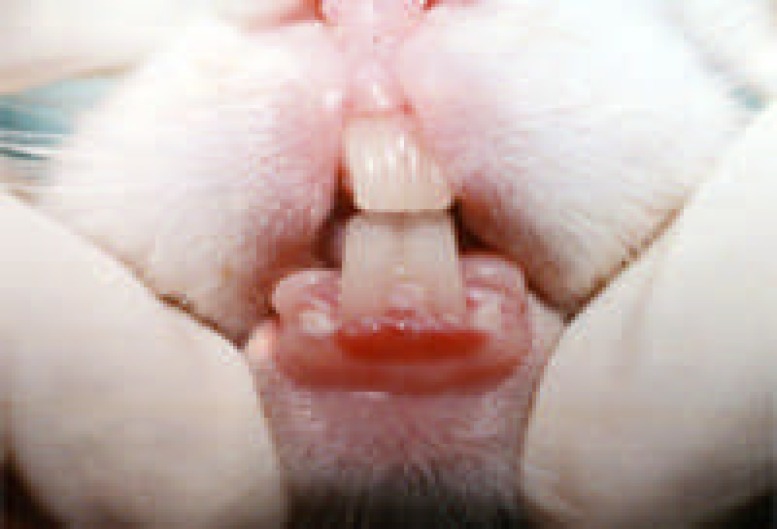
Clinical view of flap at time of suture removal (5 days group

**Figure 5:**
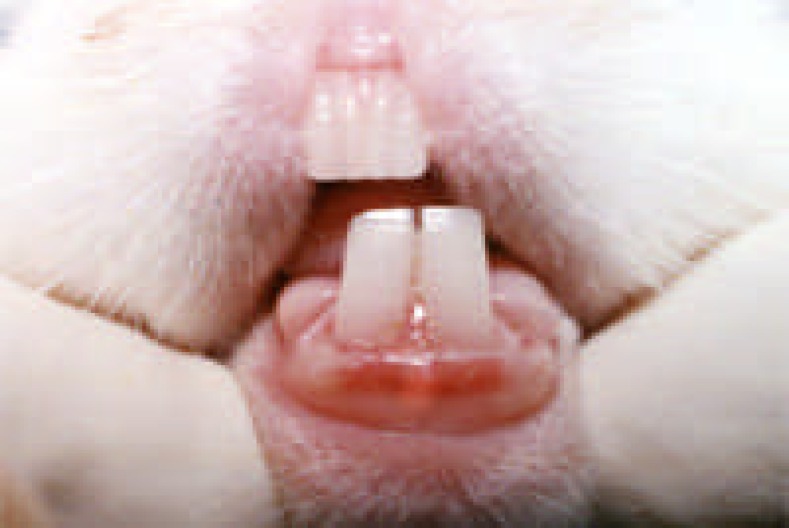
Seven days after surgery (5 days group).

**Figure 6:**
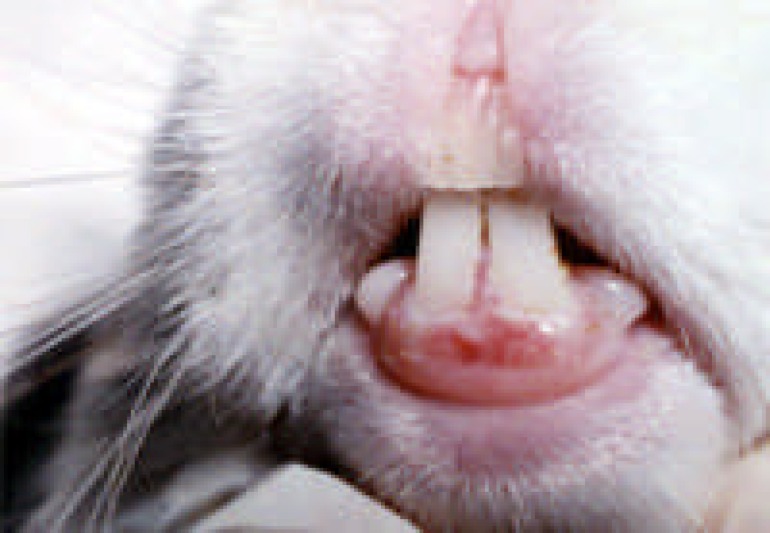
Fourteen days after surgery (5 days group).

**Figure 7:**
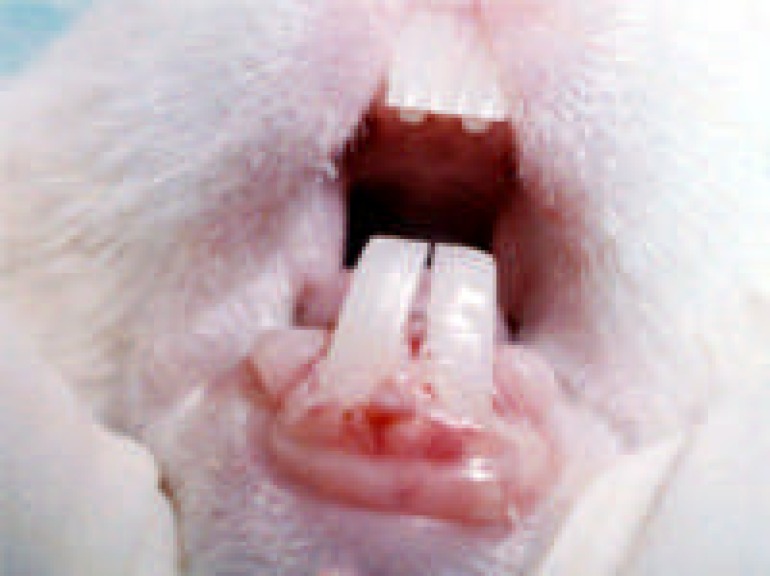
Clinical view of flap at time of suture removal (seven days group).

**Figure 8:**
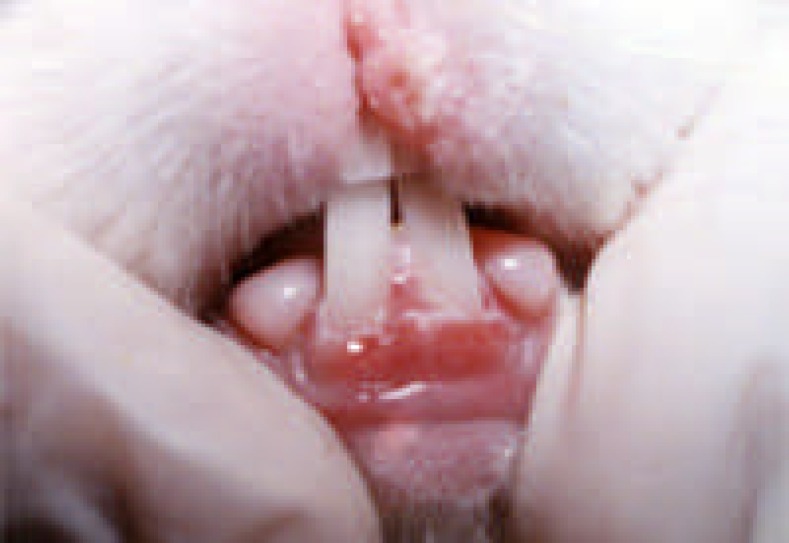
Fourteen days after surgery (seven days group).

Wound healing observation in all three groups I and II showed a significant difference between the time of suture removal, seven and 14 days after surgery in both inflammation and gingival indexes (Figure 1),(Figure 2),(Figure 3),(Figure 4),(Figure 5),(Figure 6),(Figure 7),(Figure 8) and (Table 1). Inflammation index among three groups showed that group II (five days) was significantly different in comparison with the other two groups at the day 17 (p<0.008); however, despite of less inflammation in group III, no significant difference was observed between group I and III at 14^th^ day after surgery (Table 2).

Gingival index introduced statistically significant difference in group II in comparison with both other groups at 14^th^ day after surgery (p<0.028). There was no significant difference between groups I and III although healing was much better in group III animals.

**Table1:** A comparison of gingival and inflammation indexes at the time of suture removal and 7 and 14 days after surgery in all three groups

**Group**		**Group I**	**Group II**	**Group III**
**Index**		**Mean Rank**		
GI	SRT	1	1.07	0
	7 days	2.21	1.93	-
	14 days	2.79	3	3.5
*p* value		0.001^a^	0.001^a^	0.023^b^
INI	SRT	2.86	2.93	4
	7 days	1.93	1.93	-
	14 days	1.21	1.14	0
*p* value		0. 001^a^	0.001^a^	0.001^b^

^a^: Friedman test

^b^: Wilcoxone Test

**Table 2:** A comparison among three experimental groups in gingival and inflammation index at the time of suture removal and at 7^th^ and 14^th^ day after surgery

**Group**		**Mean Rank**					***p *** **value** ^a^
**Index**		**Group I**		**Group II**	**Group III**		
INI	7 days		14.5	5.93		12.57	0.008
	14 days		14.29	7.64		11.07	0.094
GI	7 days		8.86	14.57		9.57	0.117
	14 days		6.86	15.29		10.86	*0.028*

INI: Inflammation Index;        GI: Gingival Index

SRT: Suture Removal Time;       ^a^: Kruskal wallis test

## DISCUSSION

Sutures serve to maintain tissue approximation until a wound attains sufficient tensile strength to prevent dehiscence during normal physiological activity (15). The results of recent studies have confirmed the superiority of monofilament over braided sutures in case of plaque accumulation (16-19).

It has been shown that monofilament suture materials are associated with less sever tissue response than multifilament materials such as silk suture (3). It has been stated that suture material which has monofilament structure resists fluid penetration, and thereby resulting in better healing (4).

Many clinicians, however, prefer multifilament sutures, because monofilament sutures are more difficult to manipulate, have sharp ends that irritate oral tissues, and exhibit poor knot security (20).

Polyvinylidene fluoride (PVDF) has a monofilament structure and represents an attractive suture material for vascular surgery because of its satisfactory physico-chemical properties, good biocompatibility, excellent bending properties, and low surface friction. These characteristics of PVDF sutures make these materials easy for surgeons to manipulate, particularly in terms of making a knot and sliding it into position (21).

Recently, a scanning electron microscope study has shown that plaque accumulation on PVDF suture materials was significantly different from silk sutures in oral mucosa at all time intervals of the study (19). Therefore, in this study PVDF suture material was used for flap suturing.

Gutmann and Harrison (1994) believe that the key to preventing sutures from having a negative effect on wound healing following surgery is their early removal (9). The primary purpose for placing sutures following endodontic surgery is to approximate the edges of the incisional wound to provide stabilization until the epithelium and myofibroblast-fibronectin network provide a sufficient barrier to prevent dislodgement of the flap tissues. This usually occurs within 48 hours following surgery. Therefore, it has been recommended that sutures should be removed 48 hours after periradicular surgery (4).

However, Selvig and Torabinejad (1996) in their histological study at the day 3 have detected very little stainable collagen (if there was any) and a well established fibrous union in the marginal gingiva at the day 7 (13). Selvig and Torabinejad (1996) claimed that because of previous studies (24-26) which have shown that the tensile strength of a surgical wound appears directly related to the collagen content of the granulation tissue that shows a rapid increase beginning approximately four day after wounding; it is unreasonable to remove sutures before 96 hours (13). They stated that the rapid establishment of an epithelial barrier and fibrous repair in the incisional part of the wound does not guarantee the reattachment of entire surgical flap to its bony base. In this observational study inflammation and gingival indexes showed that 5 days interval was the best time for suture removal.

It has been shown that healing wounds that are subjected to small amounts of mechanical stress, demonstrate an increase in collagen strength and formation. Excessive forces disrupt the neovasculature and collagen fibers and delay the healing (27).

It should be kept in mind that studies of periodontal wound healing have been conducted in a variety of animal spices, including humans. Spices differences in microanatomy, oral microbiota, rate of wound healing, and other factors may, thus, represent a confounding factor in interpretation of tissue reactions and in comparing results of different studies. Meanwhile many variables of wound healing, including patient nutritional status, bacterial infection, wound care and available tissue oxygen, should be researched.

## CONCLUSION

According to the findings of the present in vitro study, a five day interval can be implied as the best suture removal time in oral surgeries.
